# Capillarity in Interfacial Liquids and Marbles: Mechanisms, Properties, and Applications

**DOI:** 10.3390/molecules29132986

**Published:** 2024-06-23

**Authors:** Yang Liu, Yuanfeng Wang, John H. Xin

**Affiliations:** 1 Department of Biomedical Engineering, Sun Yat-sen University, Shenzhen 518107, China; 2 School of Fashion and Textiles, The Hong Kong Polytechnic University, Hong Kong 999077, China; yf.wang@connect.polyu.hk (Y.W.); john.xin@polyu.edu.hk (J.H.X.); 3 School of Physics and Electronic Engineering, Sichuan University of Science and Engineering, Zigong 643000, China

**Keywords:** liquid marble, liquid–liquid interface, capillarity, coalescence cascade, mass transport, microreactor

## Abstract

The mechanics of capillary force in biological systems have critical roles in the formation of the intra- and inter-cellular structures, which may mediate the organization, morphogenesis, and homeostasis of biomolecular condensates. Current techniques may not allow direct and precise measurements of the capillary forces at the intra- and inter-cellular scales. By preserving liquid droplets at the liquid–liquid interface, we have discovered and studied ideal models, i.e., interfacial liquids and marbles, for understanding general capillary mechanics that existed in liquid-in-liquid systems, e.g., biomolecular condensates. The unexpectedly long coalescence time of the interfacial liquids revealed that the Stokes equation does not hold as the radius of the liquid bridge approaches zero, evidencing the existence of a third inertially limited viscous regime. Moreover, liquid transport from a liquid droplet to a liquid reservoir can be prohibited by coating the droplet surface with hydrophobic or amphiphilic particles, forming interfacial liquid marbles. Unique characteristics, including high stability, transparency, gas permeability, and self-assembly, are observed for the interfacial liquid marbles. Phase transition and separation induced by the formation of nanostructured materials can be directly observed within the interfacial liquid marbles without the need for surfactants and agitation, making them useful tools to research the interfacial mechanics.

## 1. Introduction

Capillarity, which originated from the difference in surface tension between two immiscible phases, plays important roles in both the living and non-living processes in nature. In decades, the effects of capillary force have been widely observed in the formation of cell patterns [1], the morphogenesis of tissue [2], the formation of embryonic axes [3], and homeostasis [4], which can tune the size, morphology, structure, and function of cells and tissues through surface mechanics. Moreover, capillary force can be generated at the interfaces formed by immiscible intracellular biomolecular condensates and membranes, mediating the size, morphology, structure, and function of the intracellular organelles [5]. On the other hand, capillary forces participate in a wide range of non-living processes, such as the formation of fog [6], raindrops [7], capillary waves [8], and vortexes [9], regulating the size, shape, motion, and stability of the fluids through surface mechanics. However, the organization and morphogenesis of inter- and intra-cellular structures are mechanically complex, and involve mechanical forces with disparate origins, e.g., motor proteins [10], gravitational field [11], and hydrostatic pressure [12], other than the capillary force. Much effort has been devoted to solving the morphogenesis homologies in different species, which can be described by using the coarse-grained approach under the theoretical frames of fluid mechanics and phase transitions [13]. For example, the rigidity phase transition of the zebrafish blastoderm can be explained by analyzing the cell–cell contact topology using the percolation theory, while a few parameters, including cell–cell connectivity, cell–cell adhesion, and meta-synchronous cell cleavages, were characterized [14]. Even though the percolation model can appropriately explain the rigidity phase transition under multiple experimental disturbances such as cell fate and cell adhesion, it cannot predict the absolute value of viscosity or the changes in the force field that regulate the topological changes mechanically. To elucidate the non-linear and time-dependent mechanics in morphogenesis, it is essential to find out the basic mechanisms of the fluid mechanics involved and the functions of viscous, inertia, and capillary forces.

Capillary forces mediate the inter- and intra-cellular processes through interfacial phase separation and phase transitions, which may act as a general driving force for a wide range of interfacial phenomena, e.g., wetting, adhesion, coalescence, and Pickering emulsion. However, current measurement techniques may not support the precise in vitro or in vivo quantification of the inter- and intra-cellular capillary forces due to the difficulties in determining the strength, location, and time scale of the force. For example, the strength of the surface tension of the biomolecular condensates is estimated to be in the range of 10^−4^ N m^−1^ to 10^−7^ N m^−1^ [15,16], which is significantly weaker than the surface tension of water (*γ* = 0.072 N m^−1^) [17]. Despite the tiny scale, the capillarity in cells and tissues shares the same mechanism as the fluidics that naturally occur at the liquid–liquid interface. For example, the reorganization of genomes can be achieved by the coalescence of droplets, which are induced to form at the targeted loci of the genome [18]; the cylindrical thread formed by condensation and crosslinking of actin may break up into discontinuous pieces of tactoids driven by Rayleigh–Plateau instability [19]. Moreover, nucleolar surfactants, such as proteins NO145 and Ki-67, can adsorb and enrich at the nucleolar periphery, mediating the size of the nucleolus by impeding coalescence or Ostwald ripening [20,21]. Therefore, the capillaries at the inter- and intra-cellular interfaces can be definitely studied using the fluidic models at the visible droplet scale. Moreover, the capillary strength, location, and time scale can be measured more precisely using simple apparatuses at this scale, which may realize accurate interpretation and resolution of the unsolved mechanisms of fluid mechanics in general, such as coalescence cascade, Marangoni flow, and Stokes flow.

**Scheme 1:**
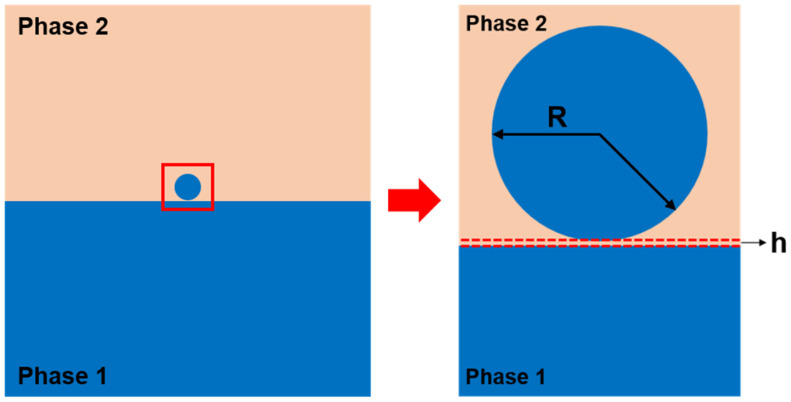
Illustration of a liquid droplet composed of the same liquid as phase 1, sitting at the liquid–liquid interface formed by two immiscible liquids. The droplet is assumed to have a spherical shape as its size is substantially small and the effect of gravity is negligible. The radius of the liquid droplet is denoted as *R*, and there may exist a thin layer of phase 2 liquid with a thickness of h between the droplet and the reservoir before the initiation of coalescence.

Interfacial liquid droplets and marbles [22], which can be formed by adding droplets at the interface between two immiscible liquid phases, can be an ideal fluidic model to study the biological capillaries. A schematic illustration of the interfacial liquid droplet is shown in Scheme 1. Specifically speaking, interfacial liquid droplets and marbles have obtained the following features, which make them outstanding in modeling the biological capillaries: (i) The phase-separated liquids mimick the phase-separated liquid-in-liquid environments existed in the cytoplasm and interstitial fluid; (ii) The viscosities of the liquids can be facilely tuned; (iii) The size of the droplet can be effectively controlled to elucidate the effect of viscous, inertia, and gravitational forces on coalescence separately; (iv) Amphiphilic surfactants with different molecular structure, size, and thermodynamics can be used to modify the droplet surface, in order to investigate the Marangoni flow at the interface and mediate the coalescence process; (v) The formation of a transparent and geometrically simple interface is advantageous for observation and modelling; the interface is concealed by the upper liquid phase as the external disturbances, such as wind, dirt, and acoustics, can be minimized.

Herein we use interfacial liquid droplets and marbles as fluidic models to elucidate the mechanisms underlying the general fluidic phenomena existing in the scale of biological capillaries, i.e., coalescence cascade, the role of surface-active particles, and mass transport at the liquid–liquid interface, which may provide useful insights for asymptotic flows and interfacial thermodynamics in biological capillaries.

The general mechanism of the coalescence cascade (CC) of a droplet has been long debated in the science community as a result of the intrinsic complexity in space and time of the process. Numerous internal and external parameters, such as droplet size, surface tension, viscosity, density, coalescence time, and the size of the daughter droplet, may mediate the CC process, affecting its stages and duration. Moreover, discrepancies in the CC phenomena at the air–liquid and liquid–liquid interfaces have been widely observed and investigated, e.g., damped CC [23], second-stage coalescence [24], migrating partial coalescence [25], and multi-stage coalescence [26], making it extremely difficult to elucidate a general mechanism. However, the central idea of the problem is how the droplet generates smaller daughter droplets during the coalescence process. Charles et al. have investigated the CC of a liquid droplet (phase 1) with a viscosity of *η*_1_ and surrounded by another liquid (phase 2) with a viscosity of *η*_2_, at the interface formed between phase 1 and phase 2 [27]. They attributed the formation of daughter droplets to the Rayleigh instability of the liquid column formed during the drainage of the droplet by the underlying liquid reservoir, and the diameter ratio of secondary to primary droplets changed with the viscosity ratio *p* = *η*_1_/*η*_2_, reaching a maximum near *p* = 1. According to their observation, no daughter droplets could form in the cases of *p* < 0.02 or *p* > 11, which was in line with the prediction of Tomotika [28], who extended the Rayleigh theory on the instability of jets [29] to the case of a cylindrical thread of a viscous liquid in the infinite mass of another viscous liquid. On the other hand, contradictory results are obtained for liquid droplet coalescence at the air–liquid interface with *p* ≫ 11. Thoroddsen et al. have observed the CC of a water droplet at the air–water interface, which can proceed for up to six steps [30]. They attributed the termination of CC to the viscous effect, which may become dominant as the Reynolds number (Re) of the droplet is sufficiently small, e.g., Re < 20. Blanchette et al. have investigated the CC of ethanol droplets at the air–ethanol interface, finding that there exists a critical Ohnesorge number (*Oh*) for droplets with small bond numbers (*Bo* < 0.2), above which total coalescence may happen [31]. Chen et al. have investigated the CC of water droplets at the interface formed by decane and water-glycerol, and the results indicate that there exists a maximum *Bo* number of droplets for CC to occur [32].

## 2. Results and Discussion

### 2.1. Mechanism of Coalescence Cascade in Interfacial Liquid Droplets

**Scheme 2:**
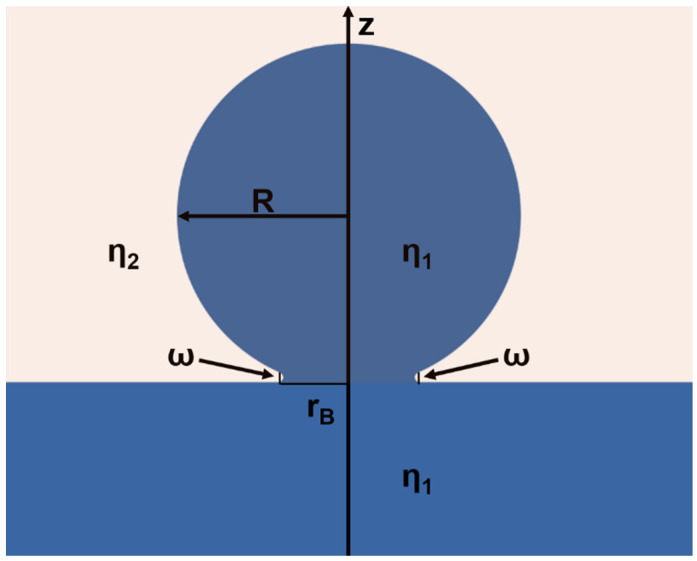
Illustration of the two-dimensional geometry of a liquid droplet with a viscosity *η*_1_ and a radius of R in the early stages of the coalescence with a liquid reservoir of the same liquid. The liquid droplet is sitting at the interface formed by the liquid reservoir and an external liquid with a viscosity of *η*_2_. The liquid bridge that joins the droplet and the reservoir has a radius of *r_B_* and a width of *ω*. The line of symmetry is represented by the *z*-axis.

From the previous research, it can be inferred that the CC of a liquid droplet on the reservoir of the same liquid can be determined by multidimensional parameters, including time (*t*), viscosity (*η*), velocity (*υ*), radius (*R*), density (*ρ*), and surface tension (*γ*). The driving force of the CC process is the mechanical potential of the liquid droplet with radius *R*, which possesses an internal pressure ΔP = 2*γ*/*R*. The Gibbs free energy of the system can be written as ΔG = −2*γ*V_mol_/*R*, where V_mol_ is the molar volume. The value of Gibbs free energy may imply the maximum work that can be conducted during the CC process; however, the actual CC process may reflect a balance between the viscous, inertial, and capillary forces, which can be essentially described by *t*, *γ*, *η*, *ρ*, and *R*. During each stage of CC, the flow from the droplet to the reservoir is driven by the formation of a highly curved liquid bridge with a length of 2π*r_B_* and a width of *ω*, which can be solved two-dimensionally [33], as shown in Scheme 2. In the 2D circumstance, the dimensionless velocity can be written as *υ* = *γ*/*η*, and the corresponding coalescence time *t* can be written as *t* = *Rη*/*γ*. Assuming the coalescence time of a liquid droplet at the air–liquid interface to be *t* and the coalescence time of the same liquid droplet at the liquid–liquid interface to be t*, there is always t* > *t* since the viscous force of the surrounding fluid exerted on the droplet surface may remarkably reduce the flow velocity, indicating liquid droplets at the liquid–liquid interface are intrinsically more stable than the liquid droplets at the air–liquid interface. However, according to our observation, the interfacial liquid droplets, which rest at the interface that composed of an upper viscous liquid and a lower liquid the same with the droplet, are much more stable compared to the existing theoretical prediction.

Specifically speaking, we investigate the CC of a water droplet at the interface formed by tetradecane (phase 2) and water (phase 1). The water droplet is generated by using a micropipette, which is subsequently released and deposited onto the tetradecane/water interface gently from a height of ~3 mm. The CC process of the droplet is recorded by a high-speed camera. To minimize the viscous and gravity forces, the size of the water droplet is set at 15 μL, corresponding to a *Bo* number of ~0.076 and an *Oh* number of ~0.003, and a precision of ca. 0.1 μL can be achieved. The radius, *Bo* number, and *Oh* number of the droplet are calculated by using Equation (1), Equation (2) and Equation (3), respectively.

(1) $$R=\sqrt[{3}]{\frac{3V}{4\pi }}$$

(2) $$Bo=\frac{\left(\rho_{1}-\rho_{2}\right)gR^{2}}{\gamma_{1}}$$

(3) $$Oh=\frac{\eta_{1}}{\sqrt{\rho_{1}R\gamma_{1}}}$$

Both of the *Bo* and *Oh* numbers are dimensional-less and are used as indicators of the importance of surface tension force against gravitational force and viscous force in general fluidic mechanics, respectively. The *Bo* number is defined as the ratio of the gravitational force to the surface tension force, and the *Oh* number is defined as the ratio of the viscous force to the surface tension force. Both of the *Bo* and *Oh* numbers are important to the CC process. For a liquid droplet of finite size, the values of its *Bo* and *Oh* numbers may determine whether partial coalescence or total coalescence will occur. For large drops where the gravitational force is dominant, the *Bo* number is the major determinant; for small drops where the viscous force is dominant, the *Oh* number is the major determinant.

**Figure 1:**
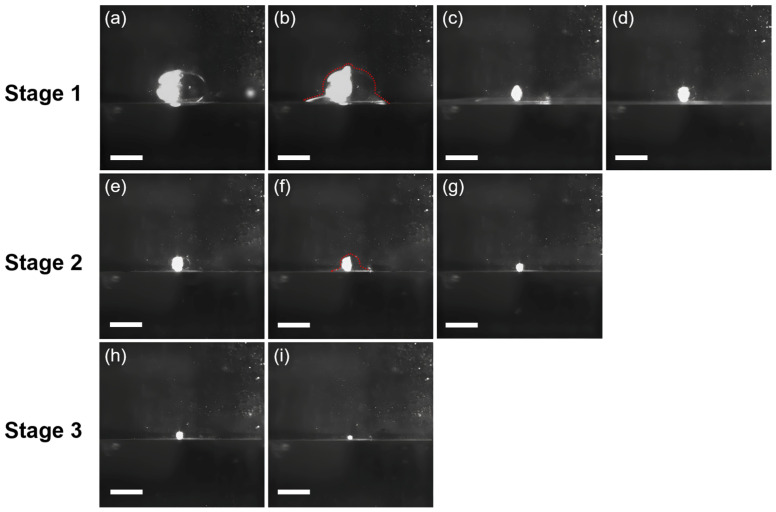
The first three stages of the coalescence cascade (CC) process of a 15 μL water droplet at the tetradecane/water interface. The first stage of CC is shown from (**a**–**d**), while the second and third stages are shown from (**e**–**g**) and (**h**,**i**), respectively. The contours of the coalescing water droplets are shown in (**b**,**f**), where the formation of liquid bridges between the droplet and the reservoir at the tetradecane/water interface can be readily observed. The time gap between each frame is 20 ms. Scale bar: 1 mm.

Typical coalescence processes of a 15 μL water droplet at the tetradecane/water interface are shown in Figure 1. When being cast at the tetradecane/water interface, the water droplet would rest at the interface as a result of the presence of a thin tetradecane layer between the bottom of the droplet and the water reservoir (Figure 1a), which is illustrated in Scheme 1 and can be a few microns thick [34]. The first step of the coalescence cascade (CC) event initiates with the formation of highly curved liquid meniscuses between the bottom of the droplet and the water surface, which are also called “liquid bridges” (Figure 1b). The liquid bridges then drive the drainage of the water droplet into the water reservoir, resulting in the widening of the meniscuses, which in turn generates capillary waves converged at the summit of the droplet and can be seen as the small protrusion at the top of the droplet (Figure 1b). Subsequently, the droplet is stretched upward, forming a liquid cylinder, while the liquid bridges quickly narrow and vanish (Figure 1c), leaving a daughter droplet with a smaller size resting at the interface (Figure 1d). In stage 1, it takes ~60 ms for the original water droplet to coalesce and form the daughter droplet. However, the daughter droplet may repeat the same coalescence process until fully coalescence occurs; due to the reduced size of the daughter droplet (hence enhanced ΔP), the coalescence may proceed in a faster and faster manner (Figure 1: Stage 2 and Stage 3). For example, it takes ~40 ms for stage 2 to complete and ~20 ms for stage 3 to complete. After all, a total of six stages can be observed; however, details of the rest of the stages are not recorded due to limited frame rates. Compared to the CC of water droplets at the air–water interface, the CC of water droplets at the oil–water interface shows three distinct aspects: (i) elliptical shape; (ii) significantly longer coalescence time; and (iii) no pinch-off. The shape of the 15 μL water droplet is remarkably deformed from sphericity at the tetradecane/water interface, forming an elliptical shape at equilibrium (Figure 1a). However, the sphericity of the water droplet is restored for the daughter droplets (Figure 1e,h). The phenomenon indicates that the deformation is caused by the under-liquid pressure, as water droplets with smaller sizes possess higher internal pressure to counterbalance the hydrostatic pressure exerted by the surrounding fluid (tetradecane). On the other hand, gravity is not the cause of the deformation, as the small *Bo* number of the 15 μL water droplet makes it negligible, and the surrounding tetradecane may assist in counter-balancing gravity [35]. Moreover, it can be predicted that water droplets with larger sizes may become more elliptical compared to the smaller ones.

The propagating stages of CC can be readily observed by naked eyes as the 15 μL water droplet is cast at the tetradecane/water interface (Video S1). However, when a water droplet with the same volume is cast at the air/water interface, naked eyes can only observe apparently full coalescence (Video S2). Indeed, the water droplet still goes through a CC process at the air/water interface, but at a speed much faster than that at the tetradecane/water interface. It has been reported that the coalescence time of the first CC stage for water droplets with volumes around 15 μL at the air/water interface is in the magnitude of a few milliseconds [26]. On the other hand, it may take a few tens of milliseconds for the first CC stage of a 15 μL water droplet at the tetradecane/water interface to complete, which is an order of magnitude longer than that at the air/water interface. The coalescence between a liquid droplet and a liquid reservoir can be described asymptotically by applying numerical methods to study the length scales and structures of the liquid bridge formed at the early stage of the coalescence, which was proposed by Eggers and coworkers [33]. They concluded that there existed $\omega \propto r_{B}^{\alpha }$, and $r_{B}$ can be described as a function of the coalescence time *t* as:

(4) $$r_{B}\left(t\right)\sim -\frac{\left(\alpha -1\right)}{2\pi }tlnt$$

And there is $\alpha =\frac{3}{2}$ when the external fluid obtained a finite viscosity. Based on their analysis, it can be estimated that the coalescence time of a liquid droplet may increase by a factor of 4 in the presence of an external fluid with a finite viscosity. However, the coalescence time of the water droplet at the tetradecane/water interface would be remarkably underestimated by using this model, which can be attributed to the underestimation of *α* and $r_{B}$. The fact that $\omega \sim r_{B}^{\frac{3}{2}}$ does not hold as $r_{B}\to 0$ for any external liquid of finite viscosity indicates that the Stokes equations cannot describe the asymptotic dynamics when $r_{B}\to 0$, where the surface tension force must be large enough to counterbalance the inertial force and initiate the coalescence. To better describe the flow dynamics as $r_{B}\to 0$, Paulsen et al. proposed a third inertially limited viscous regime between the inertial and viscous regimes and further formulated the boundary condition of the transition from the inertially limited viscous regime to the Stokes regime as [36]:

(5) $$Oh\propto \left|\ln\left(\frac{r_{B}}{8R}\right)\right|/\sqrt{4\pi }$$

**Figure 2:**
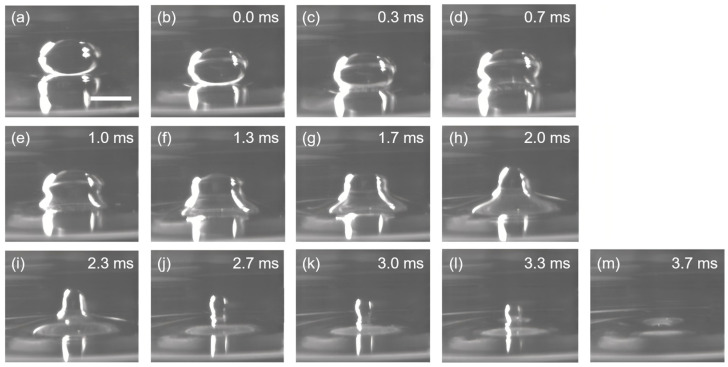
Coalescence of a viscous droplet at the tetradecane/water interface. Two distinct stages can be identified for the coalescence process: (i) The creeping stage, where the liquid bridge is growing slowly and the droplet is residing at the interface for a long duration without shape deformation, as shown in (**a**,**b**). The time gap between (**a**) and (**b**) is ~25 s. (ii) The coalescence stage, where the droplet suddenly deforms and merges into the water reservoir, leaving no trace of daughter droplets, as shown from (**b**–**m**). Scale bar: 1 mm.

They verified the inertially limited viscous regime both numerically and experimentally, showing that $r_{B}/R$ should be sufficiently large to induce the inertially limited viscous-to-Stokes transition, which can be satisfied when Oh≫1. This theory is coincident with the experimental results that the coalescence time of a 15 μL water droplet at the tetradecane/water interface is much longer than the coalescence time predicted using Stokes equations. Moreover, it is observed that the water droplets coalescence at the tetradecane/water interface do not pinch off, which can be attributed to the damping of the up-propagating capillary waves from liquid bridges to the top of the droplet by the viscous and inertial forces of the external tetradecane [23]. The existence of the inertially limited viscous regime is further evidenced by replacing the water droplet with a viscous droplet. The viscous droplet is pipetted from a viscous solution containing 0.4% polyethylene glycol 400 and 0.3% propylene glycol, and a viscosity of ~12.4 mPa·s and a surface tension of ~40 mN m^−1^ can be obtained at 25 °C. Compared to the viscosity of water (~0.89 mPa·s at 25 °C), the viscosity of the viscous droplet is one order of magnitude higher. In the meantime, it is observed that the average resident time of the 15 μL viscous droplets at the tetradecane/water interface is one order of magnitude higher than the resident time of the water droplets with the same volume (~2.8 s), which can last for ~25 s before coalescence (Figure 2a,b). However, as the length of the liquid bridge (*r_B_*) grows large enough, the coalescence of the viscous droplet happens instantaneously, and the total coalescence process finishes in 3.7 ms without leaving any trace of daughter droplets. This phenomenon indicates the transition from the inertially limited viscous regime to the Stokes (viscous) regime. The abrupt increase in the flow velocity during the transition can be explained by the cross-over of the phase boundary between the inertially limited viscous regime to the Stokes regime as *r_B_* is sufficiently large, and the creeping flow in the inertially limited viscous regime would suddenly change to Stokes flow. The velocity scaling in the two regimes can be properly described by the equations proposed by Paulsen et al. [37]:

(6) $$\upsilon_{Inertially-limited}\approx \frac{3\eta }{4R^{3}\rho }r_{B}^{2}$$

(7) $$\upsilon_{Stokes}\approx \frac{\gamma }{2\pi \eta }(\frac{r_{B}}{R})|ln(\frac{r_{B}}{8R})|$$

As a result of the relatively high viscosity, the horizontal pull at the tetradecane/water interface is restricted, and the capillary wave traveling upwards is damped, while the vertical collapse of the droplet is merely affected, as shown in Figure 2b–m. In this regard, no daughter droplets are formed, and the whole coalescence process is completed in a few milliseconds due to the high velocity of the Stokes flow.

### 2.2. Mass Transport in Interfacial Liquid Marbles at the Oil–Water Interface

**Figure 3:**
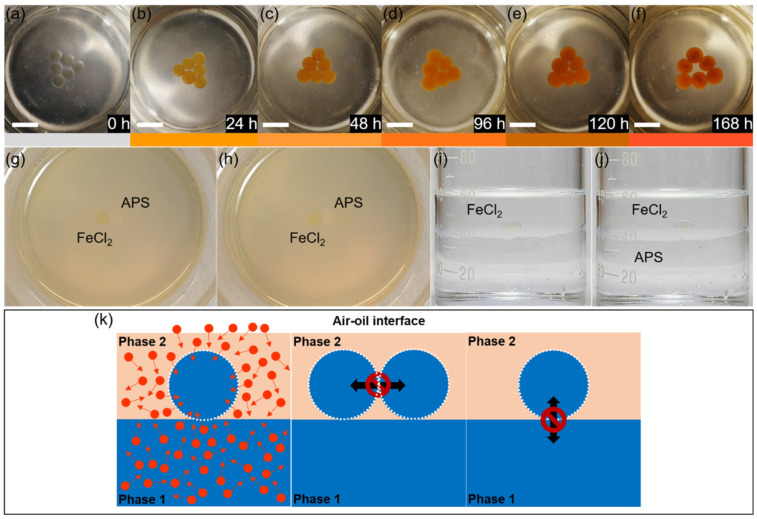
Mass transport phenomena of interfacial liquid marbles at the tetradecane/water interface. (**a**–**f**) Oxidation of an array of FeCl_2_ interfacial liquid marbles at the deoxygenated water/tetradecane interface in 168 h, images are taken at an interval of 24 h; the color bars below indicate the apparent colors of the marbles in the above images. Scale bar: 1 cm. (**g**,**h**) No liquid transport takes place between a FeCl_2_ interfacial marble and an APS interfacial marble laid adjacent to each other from 0 h (**g**) to 1.5 (**h**). (**i**,**j**) No liquid transports are observed between a FeCl_2_ marble and the underneath water phase before (**i**) and after (**j**) the addition of 1 M APS aqueous solution into the water phase. (**k**) A schematic illustration of the mass transport mechanisms of an interfacial liquid marble at the oil/water interface. The white layer represents the PVDF coating, red circles represent the gas molecules, and the arrows indicate the mass-transport directions.

To investigate the mass transport mechanism of interfacial liquid marbles, a 1 M ferrous chloride (FeCl_2_) solution is used as the liquid phase of the marbles. Interfacial liquid marbles are constructed according to reference [22]. Briefly speaking, 40 mL of deionized (D.I.) water and 20 mL of tetradecane are subsequently added into a clean 100 mL glass beaker. Bubbles and curved interfaces may form during the mixing process of the two immiscible liquid phases, which are removed and flattened using a clean glass rod, and eventually a smooth and flat interface is formed between water and tetradecane. A 30 or 60 μL droplet of 1 M FeCl_2_ aqueous solution is transferred to the surface of a hydrophobic polyvinylidene fluoride (PVDF) layer, rolling gently on the layer surface until the droplet surface is fully covered by the PVDF powders. The PVDF-coated FeCl_2_ liquid marbles show a white color, which is subsequently transferred to the tetradecane/water interface. Upon entering tetradecane, the PVDF coating quickly changes to transparent, making the original yellowish-green color of the FeCl_2_ droplet readily observable (Figure 3a). Six FeCl_2_ interfacial marbles are laid at the tetradecane–water interface, forming a triangular array. FeCl_2_ marbles are chosen as the experimental models for investigating the mass transport mechanisms at the tetradecane–water interface, as they can react with oxygen molecules and generate reddish brown Fe(OH)_3_, acting as an indicator of oxygen. While exposed to the ambient conditions, the FeCl_2_ droplets quickly change their color from yellowish-green to yellowish-brown within 24 h, and reddish-brown precipitates of Fe(OH)_3_ are readily observed at the bottom of the droplets (Figure S1a,b). Eventually the FeCl_2_ droplets become dried, leaving reddish brown precipitates (Figure S1c–f).

To evaluate the air permeability at the tetradecane/water interface, FeCl_2_ marbles are placed at the interface, where the water phase is deoxygenated prior to bubbling argon for 30 min. However, it does not prevent the oxidation of the FeCl_2_ into Fe(OH)_3_ inside the liquid marbles, as the marbles resting at the interface gradually change their colors from yellowish-green to brown and eventually reddish brown in 7 days, indicating the supply of oxygen from tetradecane and the atmosphere (Figure 3a–f). On the other hand, a slower oxidation process is observed for the FeCl_2_ marbles placed at the interface, where both tetradecane and water are deoxygenated by bubbling argon (Figure S2), compared to the control group, where both tetradecane and water are used as received (Figure S3). The mass transport between interfacial liquid marbles is evaluated by placing a 30 μL FeCl_2_ marble and a 30 μL ammonium persulfate (APS) marble at the tetradecane/water interface side-by-side without distorting the original curvature of the marbles (Figure 3g). Solid contacts are established between the surfaces of the FeCl_2_ and APS marbles, and the contacting marbles are maintained static at the interface during the experimental observation due to the presence of the protective coating layer composed of PVDF powders and the adsorbed tetradecane molecules. The APS marbles are chosen because FeCl_2_ can be readily oxidized by APS. If there is any mass transport between the APS and FeCl_2_ marbles, the color of the FeCl_2_ marble would change quickly. No mass transports are observed between the FeCl_2_ and APS marbles during the experiment (~3.5 h), and the two marbles show no observable changes during the first 1.5 h (Figure 3h). However, the color of the FeCl_2_ marble turns quickly yellow upon the injection of 30 μL of 1 M aqueous APS solution, but the color does not diffuse into the neighboring APS marble (Video S3). To evaluate the mass transfer between the water reservoir and interfacial liquid marble, a FeCl_2_ marble is placed at the tetradecane/water interface (Figure 3i), and subsequently 1 mL of 1 M APS is added into the lower water phase. No transports of APS from the water phase to the FeCl_2_ marble are observed during the experiment (Figure 3j), except the FeCl_2_ marble gradually becomes yellow after 3 h (Figure S4), as a result of the diffusion of oxygen molecules from tetradecane and the atmosphere into the marble.

From the observations of the mass transport experiments, the mechanisms of mass transport at the tetradecane/water interface can be clearly revealed: gas molecules, e.g., O_2_, can diffuse into the interfacial liquid marbles through Brownian motion; at static conditions, no liquid transport could take place between two adjacent interfacial liquid marbles, and no liquid transport could take place between the interfacial liquid marble and the underneath liquid phase. Moreover, the curvature of liquid marbles may have a significant impact on the interfacial mass transport process. Providing the *Bo* number of the marble is small and the effect of gravity is negligible, the degree of curvature of the marble is inversely proportional to its radius and size. Therefore, an interfacial liquid marble with a high curvature may have a small size, thus a high specific surface area. In other words, the high-curvature marble would have more interfacial area, where water molecules are oriented in a low-entropy tetrahedral-like structure [38], and the diffusion of molecules and ions through this water structure is impeded compared to the bulk water [39]. A schematic illustration of the mass transport mechanisms at the tetradecane/water interface is shown in Figure 3k.

### 2.3. Programmable and Versatile Microreactors Using Interfacial Liquid Marbles

Liquid marbles, which are constructed at the air–solid or air–liquid interface, have found substantial applications in miniaturized microreactors and microbioreactors [40,41,42,43], demonstrating unique features such as high interfacial area [44], oxygen permeability [45], tunable shell function [46], and stimuli-responsiveness upon applied potential and heat [47]. Compared to liquid marbles, interfacial liquid marbles may obtain extraordinary morphology and composition stability, durability, self-assembly, and transparency; the presence of the upper liquid phase may seal the interfacial liquid marbles with the ambience, prohibiting the evaporation of the encapsulated liquid and lowering the oxygen concentration at the surface of the marbles, making the marbles ideal microreactors for investigating reduction reactions.

**Figure 4:**
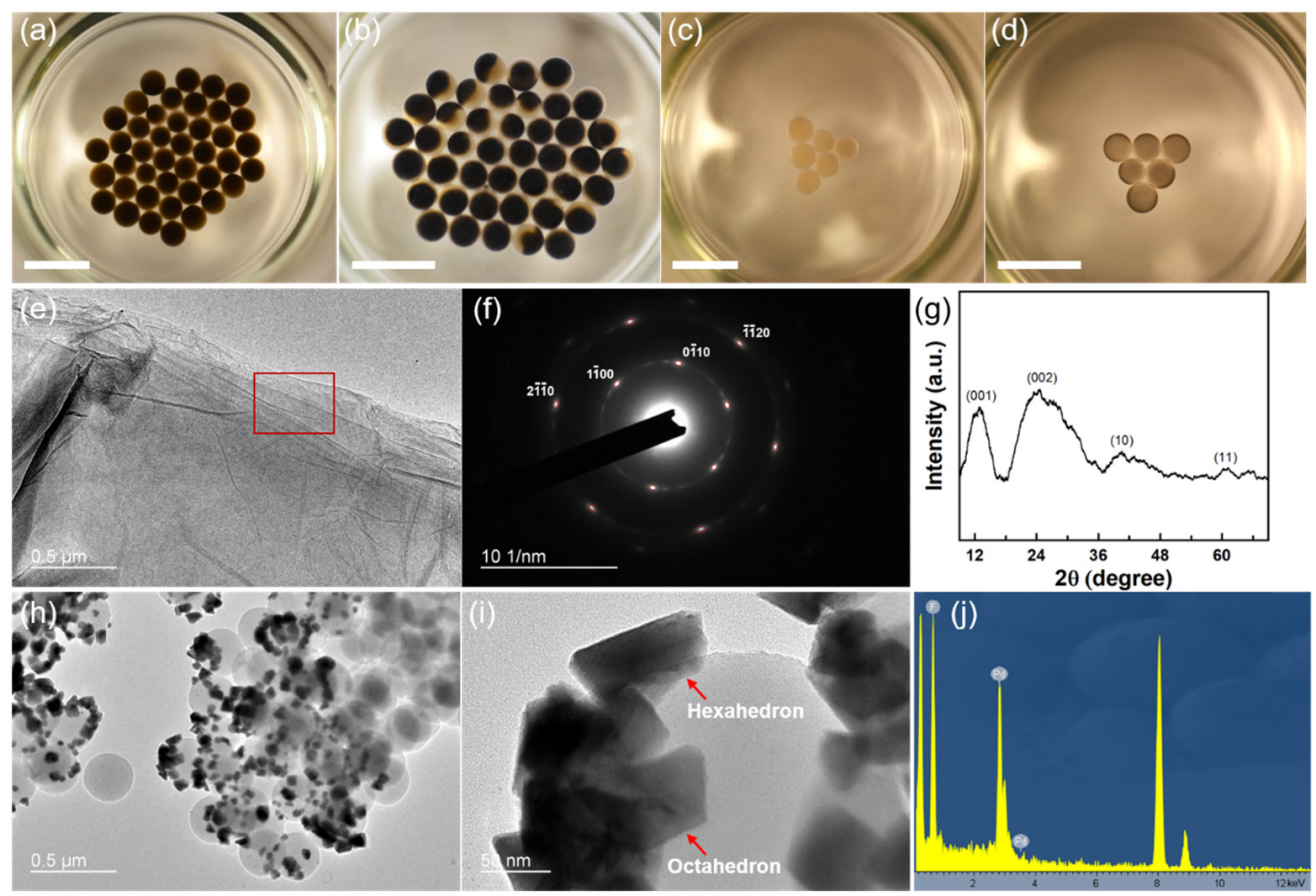
Interfacial liquid marbles as microreactors to synthesize nanomaterials with unique characteristics. (**a**) Formation of a hexagonal array of interfacial liquid marbles at the hexane–water interface by self-assembly. Each marble contains 10 µL of 3 mg mL^−1^ aqueous GO dispersion. (**b**) A hexagonal array of Janus marbles containing rGO is formed after a 24 h reduction. (**c**) Formation of a triangular array of interfacial liquid marbles at the hexane–water interface through self-assembly. Each marble contains 10 µL of aqueous 0.1 mg mL^−1^ PdCl_2_ dispersion. (**d**) Dispersed Pd nanoparticles are formed inside the marbles after an 18 h static reduction in the ambient conditions. Scale bar: 1 cm. (**e**–**g**) The TEM image, SAED pattern, and XRD spectrum of the as-obtained rGO using interfacial liquid marbles as the microreactors. The SAED pattern is taken from the red rectangular area in (**e**). (**h**) A TEM image of the Pd nanoparticles obtained from the interfacial liquid marbles showed well-dispersed Pd nanoparticles on spherical PVDF particles. (**i**) Magnified TEM image of Pd nanoparticles on PVDF, showing the coexistence of hexagonal and octahedral nanoparticles. (**j**) EDX spectrum of the Pd nanoparticles on PVDF.

Various reduction reactions can be accomplished by the interfacial liquid marbles, while state-of-the-art nanomaterials with structural and compositional uniformity can be obtained. For example, graphene and palladium nanoparticles are obtained by reducing graphene oxide (GO) and palladium chloride (PdCl_2_) in interfacial liquid marbles. For the reduction in GO, a hexagonal marble array consisting of 42 interfacial liquid marbles is fabricated on a hexane–water interface with an area of 19.5 cm^2^ (Figure 4a). Each marble contains 10 µL of 3 mg mL^−1^ aqueous GO dispersion. When being cast at the hexane–water interface, the marbles seek their positions spontaneously, driven by gravity and surface tension, eventually assembling into an ordered hexagonal array. The reduction reaction is triggered by adding 0.1 mL of hydrazine monohydrate into the lower water phase, which generates reducing hydrogen molecules through the following reaction [48]:

$$H_{2}NNH_{2}\to N_{2}\left(g\right)+2H_{2}(g)$$

The hydrogen gas can permeate into the interfacial liquid marbles and reduce the GO into rGO (reduced graphene oxide). As shown in Figure 4b, phase separation and formation of Janus marbles are observed in the interfacial liquid marbles after a 24 h reduction in the ambient and static conditions. As the hydrophilic functional groups in the GO basal planes are replaced by hydrogen during the reduction, the as-obtained rGO becomes more hydrophobic, which tends to nucleate and aggregate at the surface of the interfacial liquid marbles, causing the formation of Janus marbles. Moreover, the unique reducing environment of interfacial liquid marbles can be used to synthesize metallic nanoparticles. For example, a triangular array containing six interfacial liquid marbles is assembled at the hexane–water interface (Figure 4c). Each marble contains 10 µL of aqueous 0.1 mg mL^−1^ PdCl_2_ dispersion, which is subsequently reduced by adding hydrazine monohydrate into the lower water phase. Interestingly, no phase separations occur in the interfacial liquid marbles after an 18 h reduction, despite the color of the marbles changing from light yellow to black, as shown in Figure 4d. However, the absence of phase separation also indicates a good dispersibility of the as-synthesized metal nanoparticles in the marbles. rGO nanosheets with the coexistence of oxidized and unoxidized domains and highly dispersed Pd nanoparticles can be readily obtained using the interfacial liquid marbles as the microreactors. As shown in Figure 4e, few-layered rGO nanosheets with a planar and uniform morphology can be obtained using the interfacial liquid marbles. From the selected area electron diffraction (SAED) pattern in Figure 4f, the sharp spots corresponding to the $1\bar{1}00$-type and $2\bar{1}\bar{1}0$-type reflections of single-layer GO sheets can be readily observed with high contrast, indicating the existence of short-range order in the as-obtained rGO sheets over the length scale of a few nanometers [49]. On the other hand, the shallower ring patterns connecting the sharp spots indicate that the rGO sheets are stacking randomly with respect to each other. The XRD spectrum of the as-obtained rGO sheets shows four characteristic peaks at 2θ = 12.9°, 24.7°, 40.5°, and 60.2°, respectively (Figure 4g). The peak located at 2θ = 12.9° corresponds to the (001) reflection of the hexagonal lattice of GO, while the peak located at 2θ = 24.7° corresponds to the (002) reflection of the hexagonal lattice of rGO. Compared with ref. [50], the peak position of the (001) reflection of the as-obtained rGO shifts to higher degrees, while the peak position of the (002) reflection shifts to lower degrees, indicating a portion of GO is reduced to form rGO, accompanied by the shrinkage of the lattice structure. On the other hand, the broadening of the (002) reflection can be attributed to the large interlayer d-spacing of the rGO sheets and the intralayer micro-strains [51]. Moreover, the splitting of the (10) and (11) reflections of the graphene-like honeycomb lattice reveals the existence of a heterostructure, where oxidized and reduced domains with sizes of a few nanometers co-exist in the rGO sheets [52]. Well-dispersed Pd nanoparticles can also be obtained from the interfacial liquid marbles in ambient conditions without using any surfactants or external energies. As shown in Figure 4h, Pd nanoparticles dispersed on the surface of the spherical PVDF particles can be separated from the interfacial liquid marbles after an 18 h reduction process. The TEM image shown in Figure 4i reveals that both hexahedral and octahedral nanoparticles co-exist in the products of the reduction reaction, indicating inhomogeneous nucleation and growth processes of the nanoparticles as compared to the uniform Pd polyhedrons synthesized using capping agents [53]. The inhomogeneous growth of the Pd nanocrystals in the interfacial liquid marbles can possibly be attributed to the local hydrophobic interaction between the PVDF shell layer and Pd^0^ nuclei, resulting in spontaneous, unregulated nucleation and inhomogeneous growth by Ostwald ripening. The energy dispersive X-ray spectrum (EDX) of the nanoparticles (Figure 4j) shows that they are solely composed of Pd atoms, despite the signals from PVDF and the copper grid (peaks at 8 and 9 keV). The as-obtained Pd nanoparticles are polycrystalline, as indicated by the ringed SAED pattern (Figure S5).

## 3. Materials and Methods

### 3.1. Materials

Polyvinylidene fluoride (Kynar^®^ 761A, Arkema, Shanghai, China), tetradecane (99%, Rhawn^TM^, Shanghai, China), n-hexane (99%, Acros Organics, Shanghai, China), iron (II) chloride tetrahydrate (99%, Sinopharm Chemical Reagent, Shanghai, China), palladium chloride (99.9%, Alfa Aesar, Shanghai, China), and ammonium persulfate (98%, Sigma Chemical, Shanghai, China). All chemicals are used as received.

### 3.2. Coalescence Cascade of Water Droplets at the Tetradecane/Water Interface

Before each measurement, a micropipette (Eppendorf, Shanghai, China, 2–20 μL) is calibrated to obtain a precision of ca. 0.1 μL. The water droplet is generated by using the calibrated micropipette, which is subsequently released and deposited onto the tetradecane/water interface gently from a height of ~3 mm. The tetradecane/water interface is constructed by placing 20 mL of tetradecane on top of 50 mL of D.I. water in a precleaned glass beaker. The CC process of the droplet is recorded by a high-speed camera with a frame rate of 4000 frames per second. The temperature of the surrounding environment is ~20 °C.

### 3.3. Reduction in Graphene Oxide and Palladium Chloride in Interfacial Liquid Marbles

Graphene oxide is prepared by using a modified Hummers’ method [54]. The as-obtained graphene oxide powder is weighted and dispersed in D.I. water to make a 3 mg mL^−1^ dispersion. A total of 10 μL of the graphene oxide dispersion is fetched by using a calibrated micropipette, which is subsequently transferred to the surface of a PVDF powder layer, rolling gently on the layer until the droplet surface is fully covered by PVDF and no liquid residues are formed. The liquid marble is then loaded onto a stainless-steel lab spoon and transferred to the hexane/water interface by gently releasing it from near the surface of the liquid phase. The liquid marble would head towards the hexane/water interface under gravity while its surface became transparent due to the formation of a thin PVDF coating. Multiple liquid marbles can be cast simultaneously, which may spontaneously self-assemble into ordered patterns at the hexane/water interface, driven by the interfacial tension. Afterwards, 0.1 mL of hydrazine monohydrate is injected into the water reservoir to initiate the reduction reaction, which is allowed to proceed for 24 h at room temperature under static conditions. The reaction is terminated after 24 h, and the products inside the interfacial liquid marbles are extracted by using medical syringes for further characterization. The reduction process of PdCl_2_ in the interfacial liquid marbles is identical to that of graphene oxide, except that (i) the dispersion is obtained by 1 h sonication and (ii) the reaction time is 18 h.

## 4. Conclusions

In conclusion, interfacial liquid marbles possess unique internal and external features, making them promising models and versatile tools for the investigation of different interfacial phenomena. The extremely long life expectancy of the interfacial liquid marbles originated from the unexpectedly long coalescence time of the liquid droplet at the liquid–liquid interface, revealing the existence of a third inertially limited viscous regime when $r_{B}\to 0$. The mass transport from the droplet to the underlying reservoir can be prohibited by coating the droplet with hydrophobic or amphiphilic particles. While the transport of liquid mass is prohibited, the interfacial liquid marbles are permeable to gases. Moreover, the oxygen density at the surface of the interfacial liquid marbles is much lower than that at the air–liquid interface, making the marbles a green and cost-effective solution for conducting reduction reactions. After all, the miniaturized, transparent, versatile, and long-lasting interfacial liquid marbles may find important roles in a wide range of applications, from fluid physics to microreactors to biomolecular condensates.

## Data Availability

The original contributions presented in the study are included in the article/Supplementary Material, further inquiries can be directed to the corresponding author.

## Supplementary Materials

The following supporting information can be downloaded at: https://www.mdpi.com/article/10.3390/molecules29132986/s1, Figure S1: Oxidation and evaporation of aqueous FeCl_2_ droplets in the ambient conditions; Figure S2: Oxidation of the FeCl_2_ interfacial liquid marbles at the deoxygenated tetradecane/water interface; Figure S3: Oxidation of the FeCl_2_ interfacial liquid marbles at the pristine tetradecane/water interface; Figure S4: Photograph of the FeCl_2_ interfacial liquid marble sitting at the tetradecane/water interface with the addition of ammonium persulfate; Figure S5: TEM image and SAED pattern of the as-synthesized Pd nanoparticles. Video S1: Coalescence cascade of a 15 μL water droplet at the tetradecane/water interface; Video S2: Coalescence of a 15 μL water droplet at the air/water interface; Video S3: Color change of a 30 μL FeCl_2_ marble upon injecting 30 μL 1 M APS solution.
