# Supplementary material for: Capillarity in Interfacial Liquids and Marbles: Mechanisms, Properties, and Applications
Source: Molecules. 2024 Jun 23;29(13):2986. doi: 10.3390/molecules29132986 (PMC11243323; doi:10.3390/molecules29132986)
Supplement: Supplementary file 1 [file molecules-29-02986-s001.zip › Supporting information.pdf]

Supporting Information

Capillarity in Interfacial Liquid Marbles: Mechanisms, Properties, and Applications

Yang Liu, Yuanfeng Wang, John H. Xin

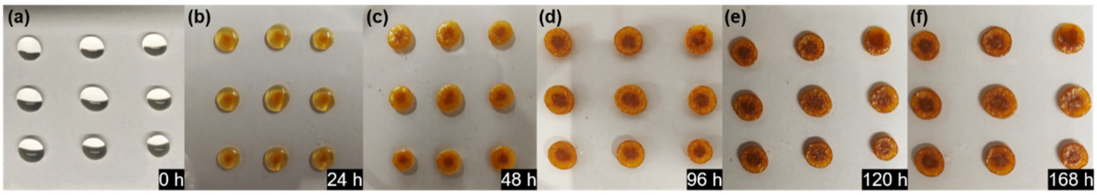

Figure S1. (a-f) Oxidation and evaporation processes of a 3×3 array of 60 μL $FeCl_2$ droplets in the ambient conditions.

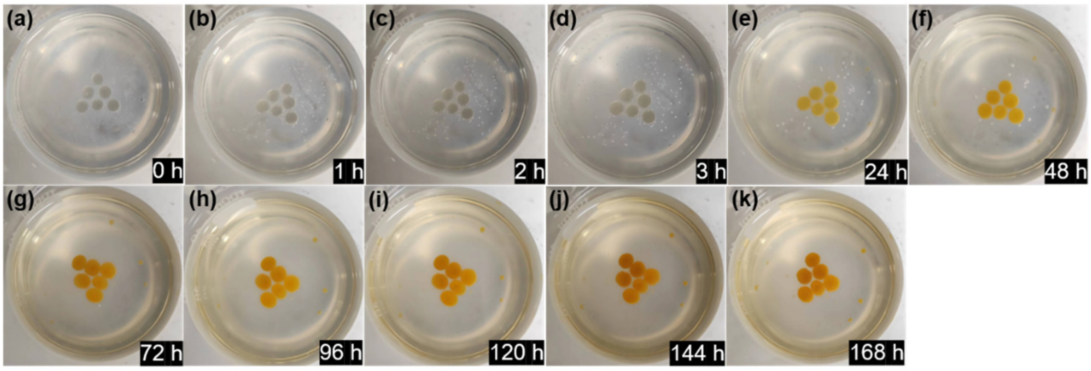

Figure S2. (a-k) Oxidation of a triangular array of $FeCl_2$ interfacial liquid marbles at the tetradecane/water interface from 0-168 h. Both of the tetradecane and water phases are deoxygenated by bubbling argon for 30 minutes.

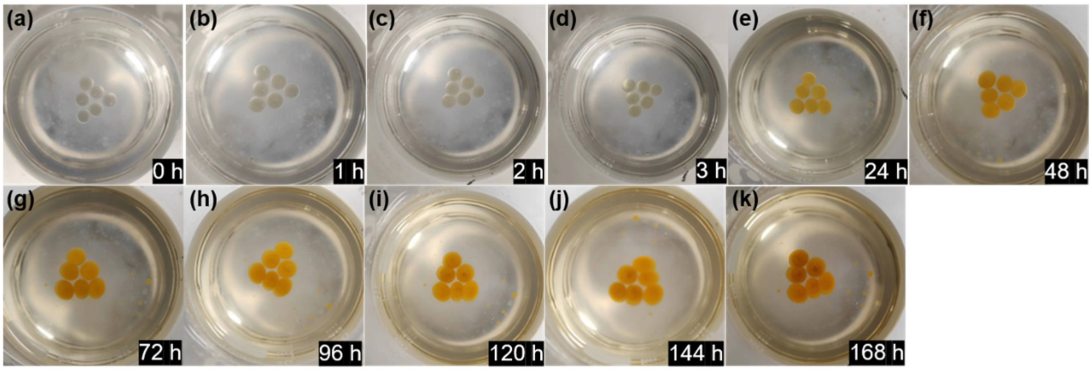

Figure S3. (a-k) Oxidation of a triangular array of $FeCl_2$ interfacial liquid marbles at the tetradecane/water interface from 0-168 h. Both of the tetradecane and water phases are used as received.

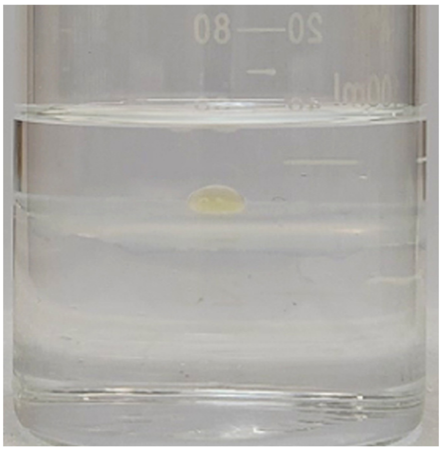

Figure S4. Photograph of a 60 μL $FeCl_2$ interfacial liquid marble sitting at the tetradecane/water interface after 3 h. 1 mL of 1 M ammonium persulfate (APS) is added into the lower water phase at the beginning.

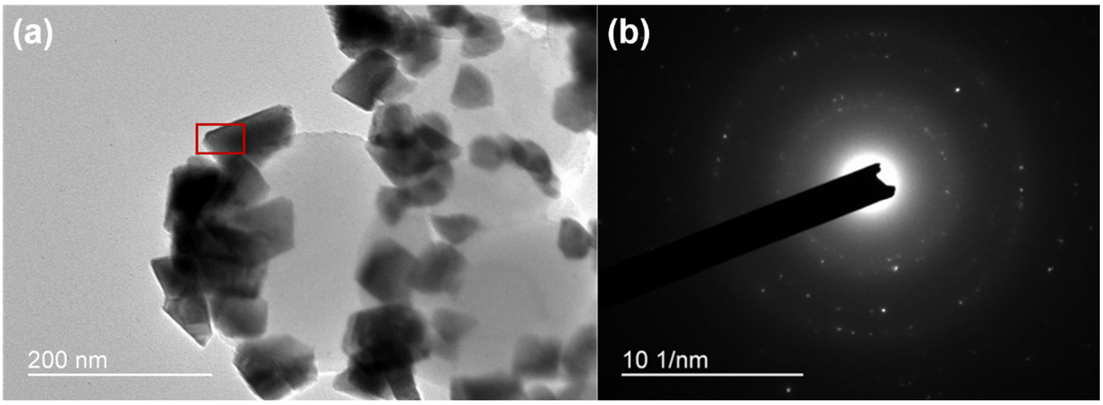

Figure S5. (a) TEM image and (b) SAED pattern of the as-synthesized Pd nanoparticles. The SAED pattern is taken from the rectangular area shown in (a).
