# Supplementary material for: Capillarity in Interfacial Liquids and Marbles: Mechanisms, Properties, and Applications
Source: Molecules. 2024 Jun 23;29(13):2986. doi: 10.3390/molecules29132986 (PMC11243323; doi:10.3390/molecules29132986)

Table of Contents

Unexpected long resident time is observed for water droplets with small Bond numbers at the oil/water interface during coalescence cascade, indicating the existence of a third inertially limited viscous regime. By coating water droplets with PVDF powder, interfacial liquid marbles with high transparency, gas permeability and unlimited durability are fabricated at the oil/water interface, which can be used as microreactors for green synthesis of nanostructured materials.

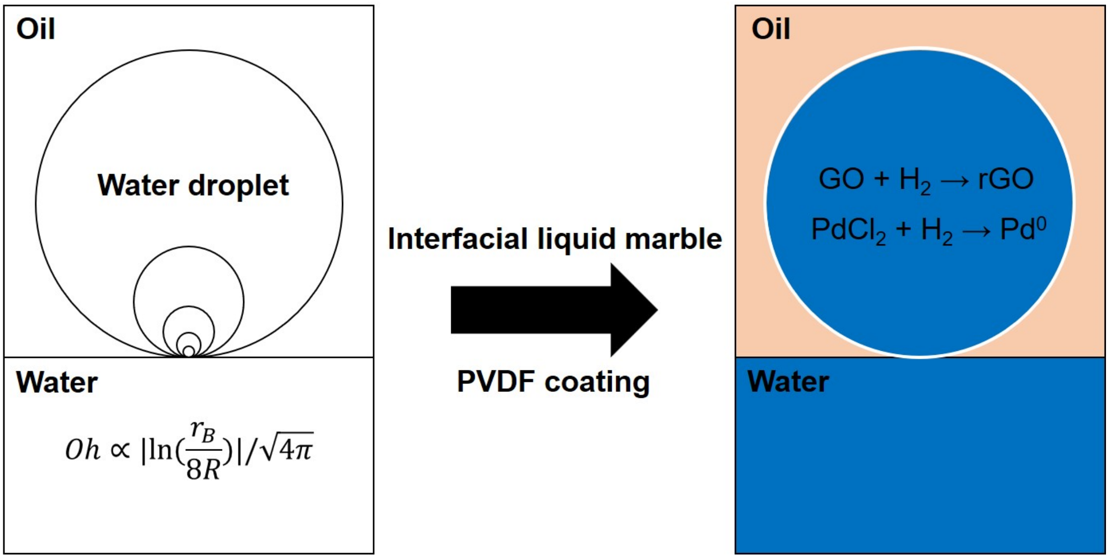

Supplement: Supplementary file 1 [file molecules-29-02986-s001.zip › Table of Contents.pdf]
